# Vitamin D Enhancement of Adipose Biology: Implications on Obesity-Associated Cardiometabolic Diseases

**DOI:** 10.3390/nu17030586

**Published:** 2025-02-06

**Authors:** Mi-Jeong Lee

**Affiliations:** Department of Human Nutrition, Food and Animal Sciences, University of Hawaii at Manoa, Honolulu, HI 96822, USA; leemj7@hawaii.edu

**Keywords:** vitamin D receptor, white adipocytes, brown adipocytes, adipogenesis, adipokines, lipid metabolism, inflammation

## Abstract

Vitamin D is activated into 1α,25(OH)_2_D through two hydroxylation steps that are primarily catalyzed by 25-hydroxylase in the liver and 1α-hydroxylase in the kidneys. The active form of vitamin D regulates myriads of cellular functions through its nuclear receptor, vitamin D receptor (VDR). Vitamin D metabolizing enzymes and VDR are expressed in adipose tissues and vitamin D regulates multiple aspects of adipose biology including the recruitment and differentiation of adipose stem cells into adipocytes and metabolic, endocrine, and immune properties. Obesity is associated with low vitamin D status, which is thought to be explained by its sequestration in large mass of adipose tissues as well as dysregulated vitamin D metabolism. Low vitamin D status in obesity may negatively impact adipose biology leading to adipose tissue dysfunctions, the major pathological factors for cardiometabolic diseases in obesity. In this review, the current understanding of vitamin D metabolism and its molecular mechanisms of actions, focusing on vitamin D–VDR regulation of adipose biology with their implications on obesity-associated diseases, is discussed. Whether improving vitamin D status leads to reductions in adiposity and risks for cardiometabolic diseases is also discussed.

## 1. Introduction

Vitamin D is obtained through diet or synthesized from cholesterol in the skin. Limited numbers of foods contain vitamin D and skin synthesis is affected by multiple factors. Therefore, vitamin D insufficiency is prevalent even in developed countries. Vitamin D is activated into 1α,25 dihydroxyvitamin D [1α,25(OH)_2_D], a seco-steroid hormone, through two hydroxylation steps that occur primarily in the liver and kidneys. The active form binds to vitamin D receptor (VDR) and regulates myriads of biological processes including its well-known actions in calcium homeostasis and musculoskeletal health [1,2]. Other organs, including adipose tissues, can also locally activate 1α,25(OH)_2_D, which regulates their functions through paracrine, intracrine, and autocrine manners [3,4].

Numerous observational studies found low vitamin D status in obesity and its associated diseases. However, whether vitamin D insufficiency predisposes individuals to obesity and cardiometabolic diseases or repletion of vitamin D leads to a reduction in adiposity and risks of cardiometabolic diseases has not been clearly demonstrated. I review the current understanding of vitamin D metabolism and vitamin D regulation of adipose biology with molecular and cellular mechanisms that could explain the inverse association between vitamin D status and obesity and cardiometabolic diseases. Moreover, findings from randomized clinical trials (RCTs) that assessed the effects of vitamin D on cardiometabolic diseases are discussed.

## 2. Methods

Pubmed, Web of Science and Cochrane Library are used as databases to search the relevant works of literature. The effects of vitamin D supplementation on markers of adiposity, serum levels of adipokines and cytokines, makers of glucose–insulin metabolism, type 2 diabetes, and cardiovascular diseases, are discussed to address how vitamin D supplementation impacts the risks of cardiometabolic diseases. For this, recent meta-analysis and systematic reviews of RCTs that have been published after our previous reviews in 2020 [5] are mainly covered.

## 3. Obesity and Adipose Tissues

### 3.1. Obesity Status

Obesity is a serious and costly chronic disease. Obesity incidence has doubled in more than 70 countries from 1980 to 2015 [6] and more than half of the global population is expected to be overweight or obese by 2035 [7]. Obesity increases risks for cardiometabolic diseases including insulin resistance (IR), beta-cell dysfunction, type 2 diabetes [T2D], metabolic dysfunction-associated steatotic liver disease, and atherosclerotic cardiovascular diseases (CVDs) that collectively increases all-cause mortality [8]. Obesity poses a substantial economic burden, accounting for USD 173 billion in medical expenditures in 2019 US dollars [9], and its impact on the global economy is expected to further increase reaching USD 4.32tn by 2035 [7]. Therefore, there is utmost interest in developing therapeutic approaches for obesity and its associated diseases.

### 3.2. Heterogeneities Among Adipose Tissues

Obesity is a status of excess fat mass and expansion of adipose tissues. Adipose tissues are located in multiple places in humans and heterogeneities in their developmental origins, functional properties, and contributions to cardiometabolic diseases have been well-documented [10,11]. Adipose tissues are divided into white and brown depots. White fat is classified into intraabdominal and subcutaneous fat depots and subcutaneous adipose tissues are further divided into the upper- and lower-body depots. While visceral adiposity independently contributes to the risks of cardiometabolic diseases, lower-body subcutaneous fat is known to be protective [10,11].

Brown adipose tissues are present in adult humans [12]. Furthermore, different types of adipocytes are present within a depot and phenotypes of adipocytes are plastic such that white adipocytes can be remodeled into brown-like cells or vice versa [13]. Compared to the white counterpart, brown and brown-like adipocytes have higher mitochondrial density and express uncoupling protein 1 (UCP1) with the potential to dissipate energy as heat [14]. The amount and activity of brown and brown-like adipocytes are decreased in people with obesity and cardiometabolic diseases, and their enhancement is thought to provide benefits [12].

### 3.3. Adipose Tissue Dysfunctions in Obesity

Adipose tissues are metabolic, endocrine, and immune organs that play crucial roles in maintaining systemic metabolic health. Adipocytes are the major storage site of excess energy, where energy is stored as triacylglycerol (TAG) in lipid droplets [15]. In response to increased systemic energy demands, TAG is hydrolyzed and fatty acids (FAs) and glycerol are released from adipocytes [16]. Adipose tissues also function as endocrine and immune organs secreting a myriad of hormones and cytokines including leptin, adiponectin, omentin, interleukin-6 (IL-6) and tumor necrosis factor-alpha (TNFα) [17]. With the development of obesity, adipose tissues become dysfunctional, exhibiting chronic inflammation, fibrosis, and hypoxia [18]. High levels of FAs, glycerol, and proinflammatory cytokines, along with reduced amounts of adiponectin and omentin released from dysfunctional adipose tissues are considered the major pathological factors increasing risks of cardiometabolic diseases in obesity [19]. These lead to impairment of insulin signaling pathways, increased systemic inflammation, and ectopic deposition, and contribute to derangement in systemic metabolic health [17].

Several factors, including oxidative stress, endoplasmic stress, fibrosis, and inflammation, have been shown to cause adipose dysfunctions in obesity [18]. Additionally, a chronic positive energy balance causes hypertrophy and death of existing adipocytes, which results in the recruitment and activation of immune cells and the exacerbation of inflammation in adipose tissues [20,21]. A careful time course study in mice showed that adipocyte death coincides with derangements in systemic metabolism during diet-induced obesity [22]. This study clearly indicates the importance of maintaining functional adipose tissue in systemic health. While what triggers adipocyte death in obesity is not clear, one hypothesis posits that adipocyte size is an important determinant [23]. Adipocyte sizes are mainly determined by TAG turnover and factors that favor lipid catabolism could reduce mechanical stress on the adipocyte and their death. Additionally, replacement of hypertrophic dysfunctional adipocytes through recruitment and differentiation of ASCs may also reduce stress on adipocytes, which contributes to the maintenance of adipose and systemic health [24].

## 4. Vitamin D Metabolism and Actions

### 4.1. Vitamin D Metabolism

Few foods naturally contain vitamin D [D_3_ (cholecalciferol) in animal-based foods or D_2_ (ergocalciferol) in plant-based foods] and multiple foods are fortified with vitamin D [25]. Oily fish and fish oils are among the best sources and other food sources include organ meat, egg yolk, and cheese [26]. Mushrooms also provide variable amounts of vitamin D_2_ and UV light treatment has been used to increase its content [27]. While bioavailability of vitamin D among different foods is not well-known [28], D_3_ is known to be more potent than D_2_ [29].

**Figure 1:**
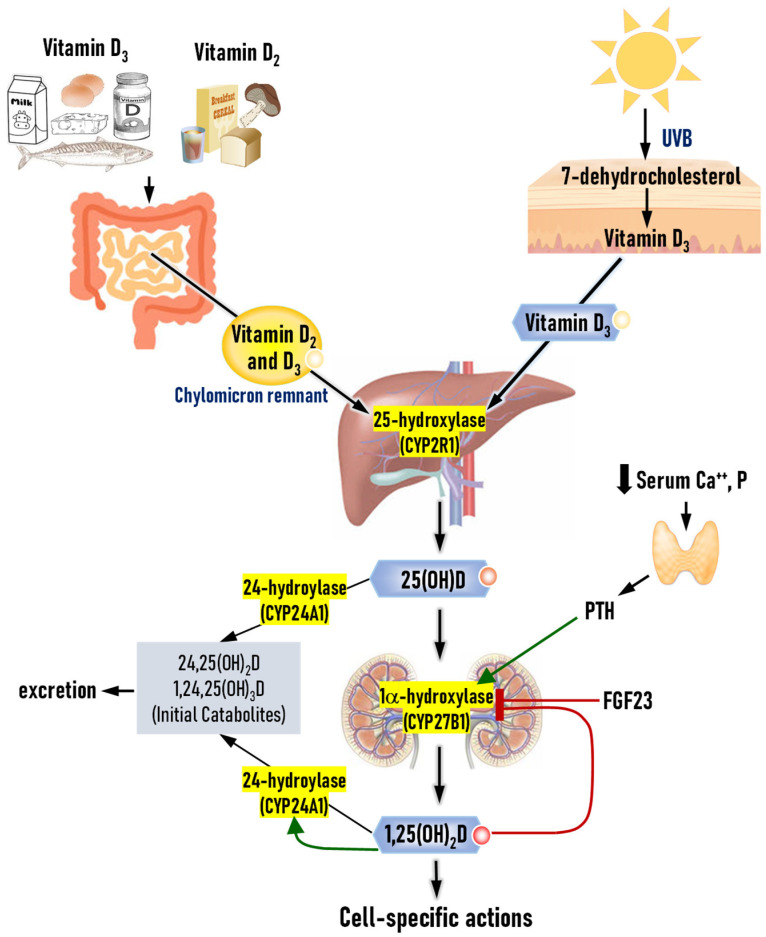
Vitamin D metabolism in the human body. Vitamin D (D_2_ or D_3_) absorbed from diets is packaged into chylomicron in enterocytes and then, secreted into the lymph. After delivering lipid-soluble nutrients to non-hepatic tissues, chylomicron remnants are uptaken into the liver. Skin-produced D_3_ binds to the vitamin D binding protein or albumin and is transported through blood. Vitamin D undergoes two hydroxylation reactions and is activated into 1,25(OH)_2_D, which are mainly catalyzed by hepatic 25-hydroxylase and nephric 1α-hydroxylase. Both 25(OH)D and 1,25(OH)_2_D are catabolized by 24-hydroxylase and further metabolized for excretion. Parathyroid hormone (PTH), secreted in response to low serum levels of calcium and phosphorus, induces 1α-hydroxylase while fibroblast growth factor 23 (FGF23) and 1,25(OH)_2_D suppress its expression. 1,25(OH)_2_D also induces the catabolizing enzyme (24-hydroxylation) expression. Through these mechanisms, 1,25(OH)_2_D levels are tightly regulated.

Ingested vitamin D is incorporated into micelles and then absorbed into the enterocytes, where it is packaged into chylomicrons and secreted into the lymph (Figure 1). Fat malabsorption conditions and obesity surgeries can therefore interfere with vitamin D absorption, causing low vitamin D status. After delivering lipid soluble nutrients to non-hepatic tissues, chylomicron remnants are uptaken into the liver. Vitamin D_3_ is also synthesized in the skin upon UVB photon-mediated photolysis of 7-dehydrocholesterol to previtamin D_3_ and its isomerization into vitamin D_3_. Skin synthesis is known to provide the majority of vitamin D in adults and approximately 5–30 min of daily or twice per week sun exposure to the face, arms, hands, and legs without sunscreen produces a sufficient amount [30]. However, multiple factors, including skin pigmentation, use of sunscreen, clothing, season, and aging, affect vitamin D synthesis and it is difficult to provide guidelines on sun exposure. Additionally, sun exposure causes photo aging and increases risks of skin cancer.

Vitamin D is converted into 1α,25(OH)_2_D through two hydroxylase steps. 25-hydroxylase (*CYP2R1*, *CYP27A1*, *CYP3A4* and *CYP2J2*) is mainly expressed in the liver while 1α-hydroxylase (*CYP27B1*) is primarily expressed in the kidneys [31]. These hydroxylases are also expressed in other organs including adipose tissues and this locally activated 1α,25(OH)_2_D also regulates their functions [3,32]. 24-hyroxylase (*CYP24A1*) converts 25(OH)D and 1α,25(OH)_2_D into 24,25(OH)_2_D and 1α,24,25(OH)_3_D, respectively, which are further metabolized and excreted into bile. Parathyroid hormone increases 1α-hydroxylase while fibroblast growth factor-23 decreases its expression. 1α,25(OH)_2_D increases 24-hydroxylase while suppressing 1α-hydroxylase [33]. Through these mechanisms, 1α,25(OH)_2_D levels are tightly regulated.

### 4.2. Vitamin D Inadequacy in Obesity and Cardiometabolic Diseases

Blood 25(OH)D levels are thought to most accurately reflect vitamin D status. While there is no consensus on cut-offs and reference intervals, most guidelines define optimal serum levels of 25(OH)D between 100 and 200 nmol/L (40–80 ng/mL) and vitamin D sufficiency as any values above 50 nmol/L (20 ng/mL) [30]. An expert committee of the Food and Nutrition Board (FNB) concluded that 25(OH)D levels greater than 50 nmol/L (20 ng/mL) represent sufficiency and less than 30 nmol/L (12 ng/mL) represent deficiency [34]. A very recent expert panel of the Endocrine Society, however, has not identified the 25(OH)D levels associated with vitamin D sufficiency or deficiency [35]. Levels higher than 125 nmol/L (50 ng/mL) can be related to adverse events that are related to hypercalcemia, and the safe upper intake level is set at 4000 IU/d by the FBN committee [34]. While excessive sun exposure may not cause vitamin D toxicity due to its limited thermal activation, frequent use of tanning beds is known to increase 25(OH)D levels to 150–200 ng/mL [36].

The FBN board established recommended daily allowances for vitamin D at 15 mcg (600 IU) for healthy adults based on the assumption that people receive minimal sun exposure [34]. A recent guideline from the Endocrine Society suggests vitamin D supplementation above the current recommendations for several groups including 1 to 18 year old and over 75 year old people and subjects with high risks of prediabetes, but not for healthy 19 to 74 year old adults [37]. Additionally, the panel suggests daily administration of vitamin D over intermittent use of mega doses [37].

Maintaining sufficiency through intake of food sources alone may be difficult and vitamin D inadequacy is recognized as a general health condition. A systematic review found that 37.3% of the global population had mean serum levels of 25(OH)D below 50 nmol/L in 2014 [38] and its inadequacy remained prevalent between 2000 and 2022 with 47.9% of participants having less than 50 nmol/L [39]. Vitamin D insufficiency is frequently reported, even in developed countries, with 40.4% of European individuals having less than 50 nmol/L of 25(OH)D [40]. Although, the highest values were observed in North America [38], an analysis of NHANES 2011–2014 data showed that 18% of US population had risks of inadequacy (30–49 nmol/L), with 5% having risks of deficiency (<30 nmol/L) [41].

Several groups are more likely to develop vitamin D insufficiency. Human milk provides 25 to 78 IU/L of vitamin D mostly as 25(OH)D and therefore, the American Academy of Pediatrics recommends supplementation of 10 mcg (400 IU)/d to breastfed infants until weaning [42]. Older adults are less likely to engage in outdoor activities and skin synthesis declines with aging and therefore, they are at risk of having inadequate levels [43]. People with kidney diseases, limited sun exposure, darker skin colors, and fat malabsorption conditions, and those who have undergone obesity surgeries, are also at risks of developing vitamin D insufficiency [44].

Numerous observational studies have reported low vitamin D status in obesity and cardiometabolic diseases [45,46]. Vitamin D releases from adipose tissue are slow and increase when lipids break down during weight loss [47,48]. Furthermore, the 25(OH)D contents in subcutaneous fat positively correlate with serum levels of 25(OH)D [49]. These suggest that larger amounts of adipose tissues in obesity sequester or buffer vitamin D, contributing to its low status. Skin synthesis of vitamin D is not affected, while its bioavailability is reduced in obesity [50]. Additionally, dysregulated vitamin D metabolism, low consumption, limited sun exposure, and skin reflectance may also contribute to the vitamin D inadequacy in people with obesity [5,51,52].

### 4.3. Vitamin D Receptor Mediation of Vitamin D Actions

**Figure 2:**
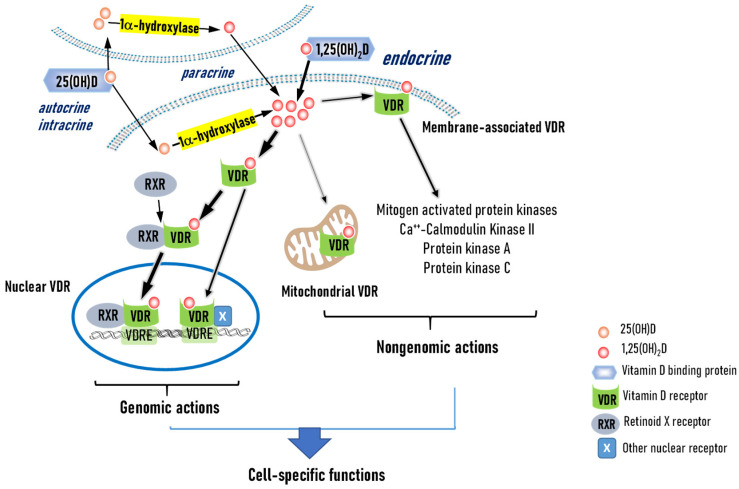
Vitamin D exerts cell-specific biological functions through genomic and non-genomic actions of vitamin D receptor. 1α,25(OH)_2_D, activated locally or delivered through blood, diffuses through the plasma membrane and binds to its nuclear receptor, vitamin D receptor (VDR). The liganded VDR forms a heteromeric complex with a retinoic acid receptor (RXR) and translocates into the nucleus. The VDR-RXR complex binds to vitamin D response elements (VDREs) in genes and regulates their transcription. VDR also regulates gene expression through interactions with other nuclear receptors (X). A fraction of VDR is present in caveolae-enriched plasma membrane and upon ligand binding, it activates multiple signaling pathways. Other actions include mitochondrial VDR regulation of mitochondrial respiration capacity.

Vitamin D exerts its actions through VDR which is expressed in muscular skeletal cells as well as non-calcium regulating cell types, including immune cells, ASCs and adipocytes [3,32]. 1α,25(OH)_2_D, locally activated or systemically delivered, diffuses through the plasma membrane and binds to a ligand-binding domain of the VDR, which then forms a heterodimeric complex with a retinoid X receptor [53] (Figure 2). The heterodimer translocates into the nucleus, associates with vitamin D response elements on target genes, and regulates gene transcription [1]. By interacting with other nuclear receptors, including nuclear factor-kappa B, VDR also affects gene transcription. More than 200 genes are known to contain vitamin D response elements and through these genomic actions, vitamin D regulates many biological functions, including calcium homeostasis, cell growth, differentiation and death, metabolism, and inflammation [2].

VDR is also present in the caveolae-enriched plasma membrane, and upon ligand binding, the membrane bound VDR rapidly activates signaling cascades that include mitogen-activated protein kinases, protein kinase A and C, and Ca^++^-calmodulin kinase II [54]. Additionally, studies have shown that 1α,25(OH)_2_D_3_ regulates respiratory capacity through mitochondrial VDR in platelets and keratinocytes [55,56]. Through these non-genomic actions, vitamin D also regulates biological processes.

## 5. Vitamin D and Adipose Tissue Biology

### 5.1. Vitamin D Metabolism in Adipose Tissues

Adipose tissues express both 25-hydroxylase and 1α-hydroxylase as well as VDR. We previously showed that their expression levels are higher in stromal vascular cells than adipocytes [3]. Furthermore, 1α,25(OH)_2_D_3_ was activated from 25(OH)D_3_ and both forms induced expression of 24-hydroxylase (*CYP24A1*), while vitamin D did not in 3T3-L1 and human preadipocytes [3,57]. These results indicate that adipose tissues can convert 25(OH)D into 1α,25(OH)_2_D, but not vitamin D to 25(OH)D. Adipose expression levels of 25-hydroxylase (*CYP2J2*) and 1α-hydroxylase (*CYP27B1*) mRNA are reduced in subjects with obesity [45]. In contrast, VDR mRNA levels in adipose tissues are higher in obesity and positively associated with inflammatory makers [45,58,59], suggesting that vitamin D metabolism and actions in adipose tissues are altered in obesity. Dysregulated vitamin D metabolism in adipose tissues of obesity may at least in part explain its reduced bioavailability, as well as the inconsistent effects of vitamin D supplementation in people with obesity.

### 5.2. Vitamin D Regulation of Proliferation and Differentiation of ASCs

1α,25(OH)_2_D_3_ suppressed proliferation in several types of preadipocyte models, including 3T3-L1 cells, Simpson–Golabi–Behemel Syndrome cells, and porcine ASCs [60,61,62]. However, the effects of vitamin D on adipogenesis remain inconclusive. While two studies showed that 1α,25(OH)_2_D_3_ suppressed differentiation of 3T3-L1 cells [63,64], another study found induction of differentiation when it was added in the absence of adipogenic induction cocktail [65]. In murine and human ASCs [3,60,66] and murine and porcine marrow-derived mesenchymal stem cells [66,67,68], 1α,25(OH)_2_D_3_ enhanced adipogenesis. VDR is also known to be involved in both pro- and anti-adipogenic actions of vitamin D [64,66,68]. These contradictory findings may be explained at least in part by differences in adipogenic programs between cell types or experimental designs [69]. More mechanistic studies are necessary to reconcile these inconsistent results.

### 5.3. Vitamin D Regulation of Adipose Tissue Metabolism

1α,25(OH)_2_D_3_ enhanced the insulin signaling pathway and insulin-stimulated glucose uptake into 3T3-L1 adipocytes [70,71]. Additionally, vitamin D induced expression of GLUT4 and insulin-stimulated glucose uptake through VDR-dependent mechanisms [68]. Vitamin D also stimulated glucose uptake into adipose tissues in mice [72]. Therefore, vitamin D may reduce glucose intolerance and IR by enhancing insulin stimulation of glucose uptake into adipocytes. Consistent with this, meta-analyses of RCTs found beneficial effects of vitamin D supplementation in people with pre-diabetes and T2D, as discussed later.

Limited information is available for vitamin D regulation of lipid synthesis and breakdown in adipocytes. 1α,25(OH)_2_D_3_ decreased de novo lipogenesis and sizes of lipid droplets, while increasing basal and stimulated lipolysis in 3T3-L1 adipocytes [73,74,75]. 1α,25(OH)_2_D_3_ also increased the expression of genes involved in FA oxidation (*PPARα*, *PGC1α* and *CPT1α*) and FA oxidation rates [74]. Additionally, studies showed that 1α,25(OH)_2_D_3_ increased UCP1 in 3T3-L1 adipocytes [73] and induced ‘brown-like’ phenotypes in human subcutaneous ASCs [76]. The central lipid droplet containing neutral lipids occupies the majority of white adipocyte volume [15] and vitamin D may reduce adipocyte sizes by increasing lipid catabolism and decreasing lipid synthesis. This could alleviate stress on hypertrophic adipocytes in obesity, reducing adipocyte death and adipose dysfunctions.

When VDR or 1α-hydroxylase (*CYP27B1*) was deleted globally, mice exhibited a lean phenotype with induction of UCP1 expression and FA oxidation in both white and brown adipose tissues [77,78]. While these findings suggest that vitamin D and VDR are involved in promotion of adiposity, their indirect catabolic actions in systemic energy metabolism may have contributed to the lean phenotypes [56,79]. Consistent with this, 1α-hydroxylase and VDR knockout mice exhibit enhanced browning of white fat as indicated by induction of UCP1 expression and appearance of multilocular cell clusters [77,78,80,81]. Conversely, adipocyte-VDR overexpressing transgenic mice exhibited lower levels of FA oxidation, UCP1 levels, and energy metabolism leading to hypertrophied adipocytes and increases in body weight [81,82]. However, female adipocyte-VDR knockout mice also had increased epididymal fat weight and hepatic fat accumulation when fed chow [83]. While the underlying causes of these seemingly inconsistent findings are not clear, sex-dependent differences in phenotypes of adipose-VDR knockout mice along with differences in diets may at least partially explain them [84]. Considering studies that have reported 1α,25(OH)_2_D_3_ decreased brown adipocyte differentiation [85] and UCP1 expression in brown adipocytes [77], more studies are necessary to fully understand how the vitamin D–VDR system affects systemic energy metabolism through regulation of brown vs. white adipocyte biology.

### 5.4. Vitamin D Regulation of Adipose Tissue Inflammation and Endocrine Functions

In both preadipocyte and adipocyte models, 1,25(OH)_2_D_3_ decreased expression of proinflammatory adipokines/cytokines (leptin, IL-6, IL-1β, IL-8, and macrophage chemoattractant protein-1), while inducing adiponectin, an anti-inflammatory and insulin-sensitizing adipokine [70,71,86,87,88,89,90,91,92]. We and others reported that VDR mediated these effects of 1,25(OH)_2_D_3_ in adipocytes [70,92]. In vivo studies in mouse models have also shown that vitamin D–VDR reduced macrophage migration into adipose tissues [90]. These suggest that vitamin D reduces adipose inflammation by acting on various cell types and this could alleviate adipose dysfunctions in obesity. Accordingly, adipose tissues from the adipocyte-VDR knockout mice exhibited increased inflammation and fibrosis when fed high-fat diet [93].

**Figure 3:**
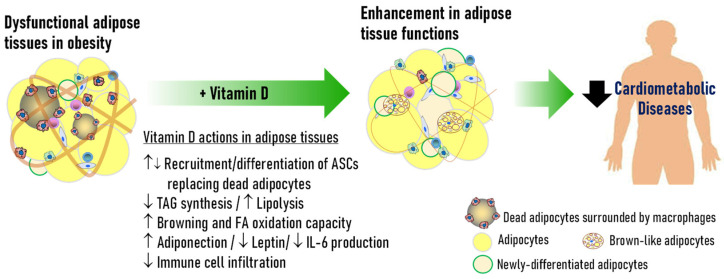
Vitamin D-mediated remodeling of adipose tissues and its impacts on cardiometabolic diseases. Vitamin D remodels adipose tissues by enhancing adipocyte development through recruitment and differentiation of ASCs, which results in replacement of dead adipocytes and increased lipid storage capacity. Vitamin D–VDR also enhances adipose tissue functions: increasing oxidative metabolism; inducing browning; decreasing inflammation; and enhancing adipose endocrine properties. Through the healthy remodeling of adipose tissues, vitamin D may decrease risk of cardiometabolic diseases.

1,25(OH)_2_D_3_ also inhibited proinflammatory signaling pathways in 3T3-L1 adipocytes [70] and immune cells [94,95]. However, several studies reported that 1,25(OH)_2_D_3_ increased expression levels proinflammatory adipokines through non-genomic actions of VDR in 3T3-L1 adipocytes [2,96]. VDR is also expressed in immune cells and vitamin D is also known to reduce adipose inflammation by acting on immune cells [97,98], supporting the anti-inflammatory actions of vitamin D and VDR. By reducing inflammation and enhancing metabolic activities in adipose tissues, vitamin D may reduce risks of cardiometabolic diseases (Figure 3).

## 6. Effects of Vitamin D Supplementation on Adiposity and Cardiometabolic Diseases

### 6.1. Vitamin D Supplementation and Adiposity

Although low vitamin D status is frequently reported in people with obesity, vitamin D supplementation did not significantly affect markers of adiposity, including body mass index (BMI), waist circumference (WC), or fat mass [99,100,101,102,103,104]. Although vitamin D plus calcium showed a small but significant reduction in body weight without any effects on BMI or fat mass [100], this was largely driven by the inclusion of the Women’s Health Initiative Calcium/Vitamin D Supplemental Trial [105]. A recent umbrella review of 14 meta-analyses, however, found a small significant effect on BMI and WC, but not on fat mass [106]. Subgroup analyses showed that vitamin D supplementation in females, Asian regions, and intervention duration ≥ 6 months significantly reduced BMI and WC [103], indicating potential benefits in specific groups and conditions.

### 6.2. Vitamin D Supplementation and Adipokines and Cytokines

The effects of vitamin D supplementation on adipokine/cytokine levels in humans remain inconclusive. While several meta-analyses showed no significant effects of vitamin D supplementation on serum adiponectin and leptin levels [107,108], another study found increased serum leptin levels in patients with T2D [109]. A more recent systematic review and meta-analysis found that vitamin D did not affect adiponectin levels in a pooled dataset, but significantly increased adiponectin in a subgroup of participants with diabetes [108]. Additionally, vitamin D had favorable effects on adiponectin when supplemented daily or co-supplemented together with calcium and the effects could be weakened by increases in BMI.

Vitamin D supplementation did not significantly affect serum levels of c-reactive protein (CRP), IL-6 and TNFα [110,111,112,113,114]. While a dosage of vitamin D supplementation was not associated with changes in inflammatory cytokine levels [112], another meta-analysis showed that ≥1000 IU/d of vitamin D had a favorable effect on CRP [111]. Additionally, age and sex affected the effects of vitamin D supplementation on CRP and IL-6 [111] and vitamin D supplementation significantly reduced CRP levels when studies in patients with T2D were exclusively considered [115]. Similarly, vitamin D supplementation reduced CRP and TNFα levels in patients with T2D [116]. Future studies should consider the confounding effects of study duration, study populations (age, sex, degree of obesity and preexisting conditions) and dosages of vitamin D supplementation.

### 6.3. Vitamin D Supplementation and Glucose Tolerance, IR and T2D

Earlier systematic review and meta-analysis showed that vitamin D supplementation did not significantly affect fasting blood glucose (FBG), fasting insulin, hemoglobin A1C (HbA1c), and homeostasis model assessment-estimated insulin resistance (HOMA-IR) index [117,118,119,120]. However, in patients with prediabetes or T2D, vitamin D supplementation significantly reduced FBG, HbA1c, fasting insulin and HOMA-IR [104,114,121,122,123,124]. Subgroup analyses showed that greater effects in nonobese than obese subjects [121,122,125], given high dosage (>2000 IU/d), individuals with vitamin D deficiency at baseline [114,123], or in patients who had diabetes < 10 years or with an age < 60 years old [124]. Vitamin D also reduced risks of T2D and increased the possibility of regression to normal glucose tolerance in prediabetic subjects [125,126]. Additionally, calcium co-supplementation promoted beneficial effects of vitamin D supplementation on FBG and HbA1c in patients with T2D [122]. Overall, these indicate the beneficial effects of vitamin D supplementation on the glucose–insulin metabolism in subjects with prediabetes or T2D.

It has been shown that maintaining serum 25(OH)D levels at least 125 nmol/L vs. 50–74 nmol/L reduced risk for T2D without any evidence of differences in the rate ratios for adverse events, including kidney stone, hypercalcemia, and hypercalciuria [126]. Subgroup analyses showed that the effects of vitamin D supplementation on diabetes control in patients with T2D were affected by weight gain, BMI, and the duration of the disease [124]. Additionally, when vitamin D was given in large doses, for a short period of time, and to patients who were non-obese, vitamin D deficient, or had optimal glycemic control at baseline, it had more prominent effects [123].

### 6.4. Vitamin D Supplementation and CVDs

Although vitamin D inadequacy is associated with higher risks of all-cause and cardiovascular mortality in patients with prediabetes or T2D, RCTs found minimal effects of vitamin D supplementation on CVD risks and outcomes [127,128,129,130]. Furthermore, a more recent systematic review on cohort studies and RCTs found that higher levels of 25(OH)D increased CVD incidence by 31% and mortality by 37% in cohort studies [131]. An umbrella review also found that vitamin D plus calcium increased the risk of stroke, which is mostly driven by calcium supplementation [132].

While these studies indicate the unfavorable effects of vitamin D supplementation on CVD, others found an improvement in blood lipid profiles, with more pronounced effects in vitamin D-deficient subjects, or in studies with a duration longer than 8 weeks, with a weekly frequency of supplementation, or that were conducted in Asia [133,134]. A recent meta-analysis also showed that vitamin D might have favorable effects on blood cholesterol and TAG levels in prediabetic and diabetic subjects with vitamin D deficiency or lower baseline BMI [124,135,136,137]. It was also reported that vitamin D (dosage greater than 800 IU/d for less than 6 months) might reduce blood pressure in subjects with ≥50 years old or in healthy subjects and patients with hypertension, without any effects in overweight and obese subjects [138]. Therefore, correcting vitamin D deficiency in certain individuals, especially those with vitamin D insufficiency, prediabetes, or T2D may provide beneficial impacts on CVDs.

## 7. Conclusions

The vitamin D and VDR system regulates multiple aspects of adipose biology, including recruitment of ASCs and their differentiation into adipocytes, as well as functional properties of adipose tissues. Contradictory results, both vitamin D promotion and inhibition of adipogenesis, are obtained from in vitro studies depending on cell types and experiment conditions used. While results from transgenic mouse models support vitamin D–VDR promotion of adipogenesis in vivo, the phenotypes of these transgenic models could be explained by altered energy metabolism. Vitamin D may also reduce adiposity through remodeling of adipose tissues that includes enhanced metabolic activities of both brown and white adipose tissues and reduced inflammation in adipose tissues. Through the healthy remodeling of adipose tissues, vitamin D may exert beneficial effects on obesity-associated cardiometabolic diseases.

Despite the well-observed low vitamin D status in obesity and cardiometabolic diseases, results from earlier RCTs showed inconsistent effects. More recent meta-analysis, however, found that vitamin D may be beneficial for improving adipokines and cytokines, markers of IR and lipid profiles in patients with prediabetes or T2D. Furthermore, data from subgroup analyses indicate that vitamin D supplementation has more favorable effects in specific populations, including people who are nonobese or have vitamin D deficiency. The reduced local activation and actions of vitamin D in addition to its sequestration in large mass of adipose tissues contribute to the lower effectiveness of vitamin D supplementation in people with obesity. Future studies should more carefully consider the confounding effects of study populations, duration and dosages of vitamin D, and co-supplementation, especially with calcium.

## Data Availability

No new data were generated.
